# Normative cardiorespiratory values in Italian first-division male soccer players: effects of age, playing position and season phase

**DOI:** 10.5114/biolsport.2025.144406

**Published:** 2024-10-25

**Authors:** Adriano Di Paco, Pierantonio Laveneziana, Paolo Onorati, Luca Trotta, Gennaro Boccia

**Affiliations:** 1 Lung Unit Casa di cura San Rossore, Pisa, Italy; 2 AP-HP, Groupe Hospitalier Universitaire APHP-Sorbonne Université, Hôpitaux Pitié-Salpêtrière, Saint-Antoine et Tenon, Service des Explorations Fonctionnelles de la Respiration, de l’Exercice et de la Dyspnée (Département R3S), F-75013 Paris, France; 3 Sorbonne Université, INSERM, UMRS1158 Neurophysiologie Respiratoire Expérimentale et Clinique, F-75005 Paris, France; 4 Presidio Tisiopneumologico e Fisiopatologia Respiratoria, Distretto Sanitario Alghero, ASL1 Sassari, ARES Sardegna, Italy; 5 San Rossore Sport Village, Pisa, Italy; 6 NeuroMuscular Function Research Group, Deparment of Clinical and Biological Sciences, University of Turin, Torino, Italy

**Keywords:** Aerobic capacity, Fitness, CPET, Football, Ventilation, Oxygen uptake

## Abstract

The study aimed to provide reference values of cardiorespiratory parameters and maximal exercise velocity (MEV) of first-division soccer players measured during a ramp exercise running test. A large (N = 741) database of cardiopulmonary exercise tests collected over a decade on Serie A teams in Italy was scrutinized. The reference values were stratified for age, playing position, and season phase. We found an average $\dot{\text{V}}{\text{O}}_{2}$peak of 4.78 ± 0.56 l/min, a relative $\dot{\text{V}}{\text{O}}_{2}$peak of 61.5 ± 5.96 ml/kg/min and an MEV of 19.0 ± 1.3 km/h. Relative $\dot{\text{V}}{\text{O}}_{2}$peak was constant from 18 to 30 years of age and then decreased, while MEV decreased constantly with increasing age. While the absolute $\dot{\text{V}}{\text{O}}_{2}$peak was similar for all playing roles (P = 0.782), the midfielders were the players with the highest relative $\dot{\text{V}}{\text{O}}_{2}$peak (P = 0.020) and they had lower body mass than the others (P < 0.001). This study presents estimates of the influence of age, player position, and season phase on $\dot{\text{V}}{\text{O}}_{2}$peak in men’s elite soccer. $\dot{\text{V}}{\text{O}}_{2}$peak values ~62 ml/kg/min fulfil the demands for $\dot{\text{V}}{\text{O}}_{2}$peak in men’s professional soccer. Midfielders showed larger relative, but not absolute, $\dot{\text{V}}{\text{O}}_{2}$peak.

## INTRODUCTION

Despite the soccer game being predominantly dependent on aerobic metabolism, it can be argued that the most decisive actions are covered by the anaerobic metabolism (i.e., sprinting, jumping, etc.). The intermittent character of a soccer game results in a succession of high-intensity activities and periods of low intensity during which the players recover [1]. The relevance of aerobic fitness has been underlined because it correlated with the total distance covered during a match [2], particularly at high intensity [3]. More recently, it has been argued that the ability to repeat high-intensity activities is only partially related to aerobic capacities [4]. Nevertheless, peak oxygen uptake ($\dot{\text{V}}{\text{O}}_{2}$peak) remains the most studied variable to determine aerobic fitness in soccer [5–8].

Maximal $\dot{\text{V}}{\text{O}}_{2}$ is typically measured during cardiopulmonary exercise testing (CPET) based on ramp running protocols. Apart from $\dot{\text{V}}{\text{O}}_{2}$peak, many other performance and respiratory measures are monitored. In terms of performance, the maximal exercise velocity (MEV), known as maximal aerobic velocity or velocity at $\dot{\text{V}}{\text{O}}_{2}$peak, serves as a valuable parameter that integrates both $\dot{\text{V}}{\text{O}}_{2}$peak and running economy into a singular metric. This metric can effectively distinguish aerobic disparities among different runners or runner groups. Unlike $\dot{\text{V}}{\text{O}}_{2}$peak or running economy in isolation, maximal exercise velocity comprehensively elucidates individual performance discrepancies.

Access to normative values is an essential component of evidencebased coaching. Normative values provide a standard against which individuals can compare their own performance and fitness. This helps set realistic goals and expectations for improvement. Understanding where an individual’s performance stands in comparison to the norm can guide the development of appropriate exercise regimens that target areas requiring improvement while capitalizing on existing strengths. Normative values are derived from large datasets, and this might be problematic when dealing with elite athletes because data on such athletes are more parsimoniously collected and shared. Moreover, the data for the Italian Serie A championship, one of the most popular in Europe, were unavailable.

The study aimed to provide reference cardiorespiratory values of first-division soccer players measured during a ramp exercise running test. Previous studies on elite soccer players [5–8] found that cardiorespiratory parameters extracted from CPET decreased with age and were influenced by playing position and season phase. Therefore, the reference values herein were stratified for age, playing position, and season phase. A large database of CPET collected over a decade on Serie A teams in Italy provided the potential for rigorous reference of the abovementioned parameters with a cohort of elite athletes.

## MATERIALS AND METHODS

### Subjects

This was an exploratory, observational, follow-up study of 451 professional male soccer players, aged between 16 and 38 years, belonging to 10 Italian “Serie A” league soccer teams. The players were tested from 1 to 5 times across the observational period; therefore there were 741 available data points. The population was examined in the period July–May of each year (from 2009 to 2017) from preseasonal training until the end of the tournament according to a standardized protocol consisting of clinical and functional assessment parameters. The clinical assessment included history of risk behaviour and physical examination, and the functional assessment included spirometry and ergospirometry. After receiving the description of the procedures and potential risks, all subjects gave their written informed consent. All procedures performed in the study complied with the ethical standards of the Internal Institutional Review Board Committee and with the 1964 Helsinki Declaration and its subsequent amendments or with comparable ethical standards. Players were unaware of the aim of the study and researchers performing analysis of results were blind to players’ identity.

### Gas measurements

For the ergospirometric test (Vmax Encore, Yorba Linda, CA, USA), we used a breath-by-breath analysis of the flows fractional inspired and expired O_2_ and CO_2_ concentrations (FiO_2_, FeO_2_, FiCO_2_, FECO_2_) obtained via mass flow and fast-responding gas analysers (fuel cell and infrared analysers). Breath-by-breath data collected during each incremental test were time-averaged over 10 s. The following variables were obtained: oxygen uptake ($\dot{\text{V}}{\text{O}}_{2}$) and its relationship to heart rate (HR), i.e. the oxygen pulse or $\dot{\text{V}}{\text{O}}_{2}$/HR, and respiratory exchange ratio (RER). Careful calibrations of flow sensors and gas analysers were performed before each measurement according to the manufacturer’s instructions. The flowmeter was calibrated with the supplied three litre calibration syringe, manually operated. The gas analysers were calibrated at three sampling points: ambient air and two pre-determined mixtures, one of 16% oxygen and 0.4% CO_2_ and the other of 26% oxygen and 0.0% CO_2_. Regarding the environmental conditions, only the air temperature was controlled (ranging from 20 to 23°C), as the tests took place in the rooms provided by the clubs.

### Procedures

An incremental symptom-limited exercise test was performed on a treadmill (Runrace 900, Technogym, Gambettola, Italy) under HR monitoring (Polar, Kempele, Finland). Subjects standing on the treadmill breathed through a mask. A continuous “ramp” protocol at constant grade (1%) (starting from 8 km/h, increasing speed by 1 km/h every 60 seconds) was used. The test was stopped when subjects complained of exhaustion. Exercise tolerance was evaluated as the maximal speed reached (maximal exercise velocity: MEV), adjusted according to a modified Kuiper’s equation: MEV (km/h) = velocity last stage completed + [*t* (s)/stage duration (s) × stage increment], where *t* is the time of the uncompleted stage expressed in seconds [9].

### Statistical analysis

The $\dot{\text{V}}{\text{O}}_{2}$peak estimates were calculated using four different RER end criteria (i.e. ≥ 1.00; ≥ 1.05; ≥ 1.10; ≥ 1.15), and the frequency of occurrence for each RER criterion was calculated. The descriptive statistics of anthropometric and physiological measurements were calculated for RER ≥ 1.00 as $\dot{\text{V}}{\text{O}}_{2}$peak did not increase with increasing RER criteria. The mean, standard deviation (SD), and values at -3, -2, -1, 0, 1, 2, 3 z scores were reported. We also reported the T scores according to previous suggestions [10, 11]. Age was stratified in four age groups: 18–20 (N = 165), 21–25 (N = 234), 26–30 (N = 222), ≥ 31 (N = 108) years of age. Tests performed before the official season were defined as pre-season, while tests performed during the official season were defined as in-season. The playing positions were defined as defender, midfielder, or forward, as found on a popular soccer website called Transfermarkt.com. We adopted Student’s T test for independent samples to test possible differences between the off-season and in-season and ANOVA to test differences between RER criteria, playing role and age.

## RESULTS

### RER criteria

The soccer players reached RER ≥ 1.00 on 727 occasions (frequency 94%); RER ≥ 1.05 on 596 occasions (frequency 77%); RER ≥ 1.10 on 366 occasions (frequency 47%); RER ≥ 1.15 on 172 occasions (frequency 22%).

**FIG. 1:**
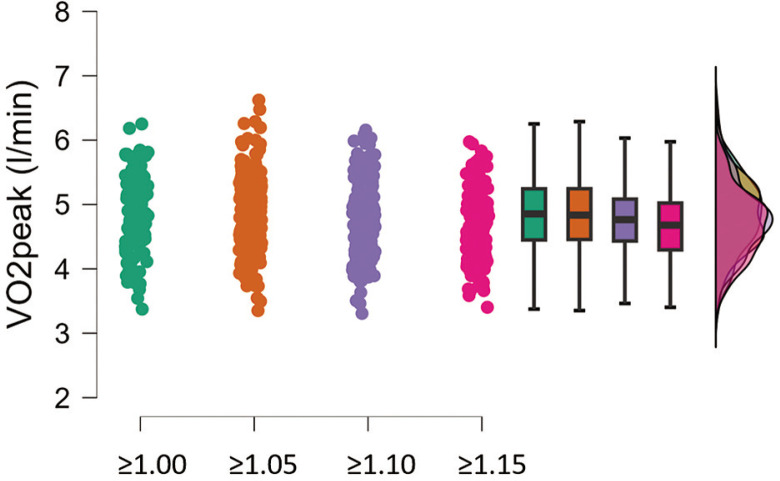
Raincloud plot, box plot and distribution of $\dot{\text{V}}{\text{O}}_{2}$peak estimates are shown for each respiratory exchange ratio (RER) criterion (i.e. 1) ≥ 1.00; 2) ≥ 1.05; 3) ≥ 1.10; 4) ≥ 1.15).

$\dot{\text{V}}{\text{O}}_{2}$peak did not change significantly with increasing RER criteria (Figure 1). There was a non-statistically significant decrease of 2.5% from RER 1.00 to RER 1.15 criteria. The average $\dot{\text{V}}{\text{O}}_{2}$peak values stratified for each RER threshold were as follows: RER ≥ 1.00: 4.78 ± 0.56 (l/min); RER ≥ 1.05: 4.76 ± 0.55 (l/min); RER ≥ 1.10: 4.71 ± 0.54 (l/min); RER ≥ 1.15: 4.66 ± 0.53 (l/min). For this reason, we applied RER ≥ 1.00 as the criterion for providing reference values.

### Normative data

**FIG. 2:**
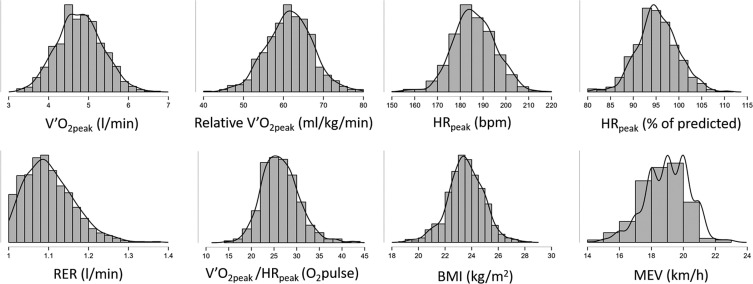
Distributions of the most relevant measurements are shown (the y-axis is expressed in arbitrary units).

The normative data calculated over 727 occasions with RER ≥ 1.00 are reported in Table 1 and Table 2. We found an average $\dot{\text{V}}{\text{O}}_{2}$peak of 4.78 ± 0.56 l/min, relative $\dot{\text{V}}{\text{O}}_{2}$peak of 61.5 ± 5.96 ml/kg/min, and an average maximum exercise velocity of 19.0 ± 1.3 km/h. The distributions of the most relevant variables are reported in Figure 2.

**TABLE 1:** Descriptive statistics of normative values

		Parameter	$\dot{\text{V}}{\text{O}}_{2}$peak (l/min)				Relative $\dot{\text{V}}{\text{O}}_{2}$peak (ml/kg/min)			

		Age (years)	≤ 20	21–25	26–30	≥ 31	≤ 20	21–25	26–30	≥ 31

		Mean	4.54	4.80	4.94	4.75	61.83	61.82	61.64	59.91

		SD	0.52	0.54	0.56	0.56	4.87	6.11	5.19	6.12
**Description**	**T score**	**Z score**									
Extremely poor	20	-3	3.48	3.34	3.35	3.54	47.77	43.76	45.59	45.22
Very poor	30	-2	3.63	3.79	3.86	3.75	52.73	50.63	49.12	47.63
Poor	40	-1	4.04	4.27	4.40	4.16	56.98	55.20	56.08	53.09
Average	50	0	4.50	4.81	4.94	4.70	61.70	61.70	61.70	60.65
Good	60	1	5.04	5.34	5.51	5.41	66.10	67.81	67.11	66.20
Very good	70	2	5.63	5.88	6.07	5.83	71.60	74.06	73.37	70.60
Excellent	80	3	6.16	6.18	6.47	6.35	76.67	77.27	79.02	75.75

**TABLE 2:** Descriptive Statistics of secondary data

Parameter	Age (years)	Mean	SD	Parameter	Age (years)	Mean	SD
Height (m)	≤ 20	1.80	0.06	HRpeak (bpm)	≤ 20	191.6	8.0
	21–25	1.82	0.05		21–25	186.4	8.8
	26–30	1.82	0.05		26–30	184.1	8.5
	≥ 31	1.81	0.05		≥ 31	181.8	9.0

Body mass (kg)	≤ 20	73.5	6.5	$\dot{\text{V}}$CO_2_peak (l/min)	≤ 20	4.97	0.59
	21–25	77.8	5.8		21–25	5.28	0.63
	26–30	80.1	6.1		26–30	5.48	0.65
	≥ 31	79.3	5.4		≥ 31	5.27	0.69

BMI (kg × m2)	≤ 20	22.6	1.3	RER	≤ 20	1.09	0.05
	21–25	23.3	1.1		21–25	1.09	0.05
	26–30	24.0	1.2		26–30	1.10	0.06
	≥ 31	24.2	1.1		≥ 31	1.11	0.07

MEV (km/h)	≤ 20	19.3	1.3	$\dot{\text{V}}{\text{O}}_{2}$/HR (O_2_pulse) peak	≤ 20	24.43	3.47
	21–25	19.1	1.3		21–25	26.68	3.81
	26–30	18.8	1.3		26–30	27.53	4.37
	≥ 31	18.6	1.4		≥ 31	26.83	3.85

### Age

**FIG. 3:**
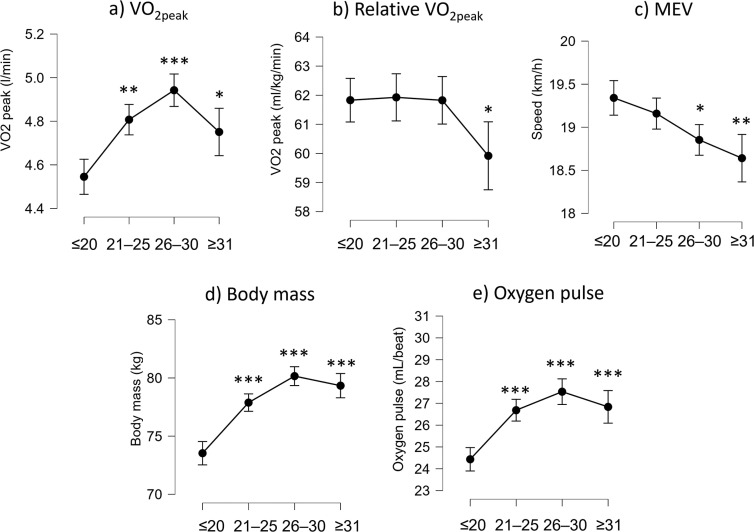
Box plot of $\dot{\text{V}}{\text{O}}_{2}$peak, relative $\dot{\text{V}}{\text{O}}_{2}$peak, maximum exercise velocity (MEV), body mass, and oxygen pulse are shown for each age group. Statistically significant differences are indicated above the box plots (*P < 0.05; **P < 0.01; ***P < 0.001).

$\dot{\text{V}}{\text{O}}_{2}$peak varied across age groups (F_(3,725)_ = 16.9, p < 0.001). It was the greatest for the players aged up to 30 years (4.9 ± 0.5 l/min, P < 0.001, Figure 3a), and was lower for players older than 30 years (4.7 ± 0.5 l/min, P = 0.016). Age had an effect on relative $\dot{\text{V}}{\text{O}}_{2}$peak (F_(3,725)_ = 3.3, p = 0.020) only for ages greater than 30 years (Figure 3b). Indeed, players older than 30 years showed lower values (59.9 ± 6.1 ml/kg/min) than younger players (≈61.8 ml/kg/min). MEV constantly decreased across age groups (F_(3,725)_ = 7.6, p < 0.001) with the youngest players showing the highest MEV (Figure 3c). Body mass was the greatest in the group aged up to 30 years (F_(3,725)_= 41.2, p < 0.001, Figure 3d). Oxygen pulse (F_(3,725)_ = 20.4, p < 0.001) was greater in players older than 20 years of age compared to those younger than 20 years of age (Figure 3e).

### Playing role

**FIG. 4:**
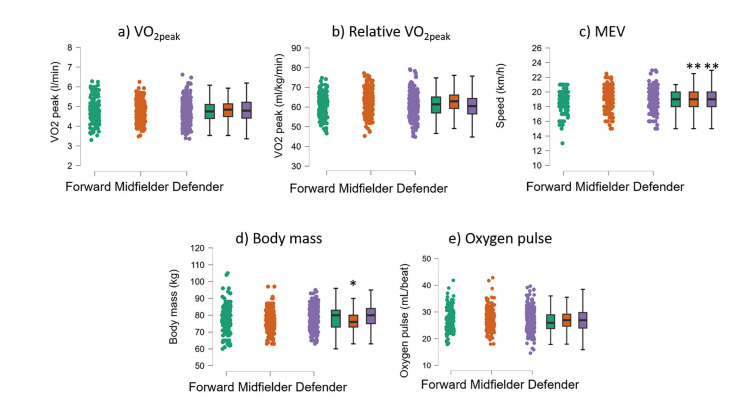
Raincloud and box plot of $\dot{\text{V}}{\text{O}}_{2}$peak, relative $\dot{\text{V}}{\text{O}}_{2}$peak, maximum exercise velocity (MEV), body mass, and oxygen pulse are shown for each playing position. Statistically significant differences are indicated above the box plots (*P < 0.05; **P < 0.01; ***P < 0.001).

Figure 4 shows the different distributions of the most relevant variables between playing roles. ANOVA showed that absolute $\dot{\text{V}}{\text{O}}_{2}$peak (F_(2,695)_ = 0.2, p = 0.782) was not different between roles (Figure 4a). Relative $\dot{\text{V}}{\text{O}}_{2}$peak (F_(2,695)_ = 9.5, P < 0.001, Figure 4b) was higher in midfielders (62.88 ± 5.63 ml/kg/min, P < 0.001) compared to forwards (61.10 ± 5.74 ml/kg/min) and defenders (60.78 ± 5.13 ml/kg/min). MEV (F_(2,693)_ = 4.9, p = 0.007) was greater in midfielders (19.1 ± 1.3 km/h) than in forwards (18.7 ± 1.4 km/h, Figure 4c). Body mass (F_(2,695)_ = 10.3, p < 0.001, Figure 4d) was lower in midfielders (76.6 ± 5.6 kg) than in forwards (78.3 ± 8.1 kg) and defenders (79.1 ± 6.2 kg). Oxygen pulse did not vary across playing roles (P = 0.406, Figure 4e).

### Pre-season vs in-season

**FIG. 5:**
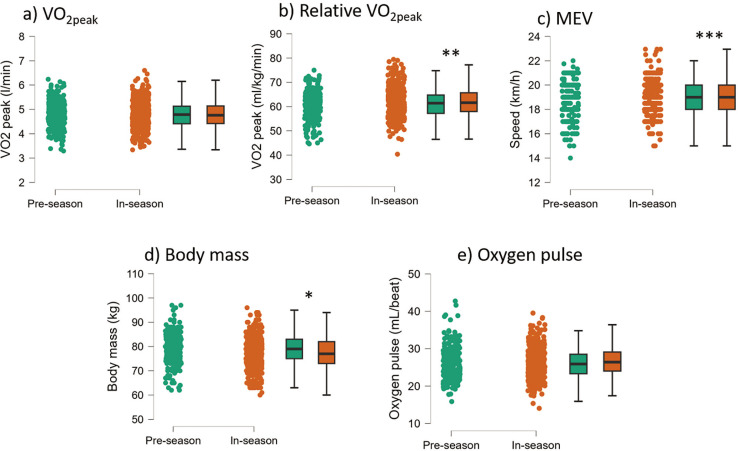
Raincloud and box plot of $\dot{\text{V}}{\text{O}}_{2}$peak, relative $\dot{\text{V}}{\text{O}}_{2}$peak, maximum exercise velocity (MEV), body mass, and oxygen pulse are shown for pre-season and in-season phases. Statistically significant differences are indicated above the box plots (*P < 0.05; **P < 0.01; ***P < 0.001).

We found minor differences when comparing in-season and preseason results (Figure 5). While absolute $\dot{\text{V}}{\text{O}}_{2}$peak did not change (P = 0.988), relative $\dot{\text{V}}{\text{O}}_{2}$peak (P = 0.006, d = 0.211) and MEV (P < 0.001, d = 0.338) increased during the in-season. Of note, there was a decrease in average body mass in season (P = 0.005, d = 0.218), while there was no change in oxygen pulse (P = 0.259).

## DISCUSSION

A database of 727 graded exercise tests was adopted to determine relevant cardiorespiratory reference values for Italian first-division soccer players. We found an average $\dot{\text{V}}{\text{O}}_{2}$peak of 4.78 ± 0.56 l/min, a relative $\dot{\text{V}}{\text{O}}_{2}$peak of 61.5 ± 5.9 ml/kg/min, and an MEV of 19.0 ± 1.3 km/h. Furthermore, we found that relative $\dot{\text{V}}{\text{O}}_{2}$peak was stable between 18 and 30 years of age (≈61.8 ml/kg/min) and then slightly decreased (≈59.9 ml/kg/min). Midfielders were the players with the highest relative $\dot{\text{V}}{\text{O}}_{2}$peak together with the lowest body mass. MEV and relative $\dot{\text{V}}{\text{O}}_{2}$peak were highest in season, while body mass was lowest in season.

The traditional standard for determining $\dot{\text{V}}{\text{O}}_{2}$peak involves reaching a point where $\dot{\text{V}}{\text{O}}_{2}$ levels stabilize even if the workload increases [12]. In cases where a plateau is not achieved, other indicators such as a certain level of RER and age-adjusted estimations of maximum HR are considered [12]. In cases of failure to achieve a plateau in $\dot{\text{V}}{\text{O}}_{2}$, RER is the most used secondary criterion for attaining $\dot{\text{V}}{\text{O}}_{2}$max. RER ≥ 1.15 is the originally recommended secondary end criterion [13]. Only 22% of the soccer players enrolled in the present study reached RER ≥ 1.15; therefore, most players would have been discarded from the analysis if considering this threshold. In the general healthy population, the frequency of attending RER 1.15 is much larger, i.e. 65% [14]. The present result comes from top-level soccer clubs, and therefore it must be taken into account in a practical setting. It means that when working with top-level soccer players one is likely to observe lower intensity of exertion than that found in other populations (e.g. general populations or athletes practising other sports). However, lower RER cut-off values have been suggested in the literature, such as 1.10 [15], ≥ 1.05 [16], or ≥ 1.0 [17]. When applying lower cut-off values, the $\dot{\text{V}}{\text{O}}_{2}$peak of the present population did not significantly change (Figure 1). For this reason, it was not necessary to apply higher thresholds consequently removing most players from the analysis. Therefore we applied RER ≥ 1.00 as the criterion for $\dot{\text{V}}{\text{O}}_{2}$peak calculation and for subsequent analysis. Nevertheless, the peak heart rate was higher than 95% of age-predicted HRmax (see Table 1), ensuring almost maximal exertion.

Normative values in exercise and sport science provide a framework for setting goals, monitoring progress, and tailoring training programmes. The present study provides the distribution (see Table 1 and Figure 2) of cardiorespiratory parameters from a large sample (451 players tested on a total of 727 occasions) of first-division Italian soccer players. We found a $\dot{\text{V}}{\text{O}}_{2}$peak of 4.78 ± 0.56 l/min and a relative $\dot{\text{V}}{\text{O}}_{2}$peak of 61.5 ± 5.96 ml/kg/min. Our findings are in line with a previous study [5] which reported an average $\dot{\text{V}}{\text{O}}_{2}$peak of 4.90 ± 0.48 l/min and a relative $\dot{\text{V}}{\text{O}}_{2}$peak of 62–64 ml/kg/min for a large sample (N = 546) of first-division Norwegian league players. In the Brazilian first division, the relative $\dot{\text{V}}{\text{O}}_{2}$peak was reported to be slightly lower, at 56.58 ± 5.03 ml/kg/min [6], while in the Croatian first division it was 60.1 ± 2.3 ml/kg/min [8]. In the Belgian first division, the reported relative $\dot{\text{V}}{\text{O}}_{2}$peak was between 55 and 62 ml/kg/min, depending on the role [7]. Overall, the present results are in line with or slightly higher than those reported in other nations, even considering that all studies’ average age was around 25 years.

The effect of age on elite soccer players’ cardiorespiratory parameters was diverse (Figure 3). Indeed, absolute $\dot{\text{V}}{\text{O}}_{2}$peak increased from 18–20 years up to 26–30 years of age (Figure 3a), possibly because of increasing body mass during the same period (Figure 3d). The concurrent increase in absolute $\dot{\text{V}}{\text{O}}_{2}$peak and body mass led to a stable relative $\dot{\text{V}}{\text{O}}_{2}$peak from 18–20 years up to 26–30 years of age. This trend highlights the importance of reporting body mass in such a context, because stable relative $\dot{\text{V}}{\text{O}}_{2}$peak might hinder different trends in cardiorespiratory absolute capacities. As expected, possibly because of increased body mass or decreased muscle strength [18] at older ages, the MEV decreased with increasing age (Figure 3c), stressing the importance of an actual measure of performance, beyond metabolic parameters, in players’ CPET.

Midfielders were the players with the highest relative $\dot{\text{V}}{\text{O}}_{2}$peaktogether with the lowest body mass. The fact that the highest relative $\dot{\text{V}}{\text{O}}_{2}$peak of midfielders was mainly due to their lower body mass rather than their higher absolute $\dot{\text{V}}{\text{O}}_{2}$peak was previously suggested by Tonnessen and colleagues [5]. Indeed, absolute $\dot{\text{V}}{\text{O}}_{2}$peak was similar among playing positions (Figure 4). However, a 2% difference in relative $\dot{\text{V}}{\text{O}}_{2}$peak cannot define them as completely different athletes from others (Figure 4). Differences in metabolic metrics are driven more by the peculiarities of the player’s position than by the physiological characteristics of the player in that position [1]. Nevertheless, because of their highest relative $\dot{\text{V}}{\text{O}}_{2}$peak, midfielders showed the highest MEV.

Soccer players in our sample reached higher peak exercise velocity in the in-season than pre-season (Figure 5). This is in line with previous studies [5], and it is an expected result. The tests were conducted after at least four weeks of soccer-specific training. However, the increase in maximal exercise velocity was not due to the rise in absolute $\dot{\text{V}}{\text{O}}_{2}$peak (which remained constant) but to the decrease in body weight, which resulted in higher relative $\dot{\text{V}}{\text{O}}_{2}$peak. The constant absolute $\dot{\text{V}}{\text{O}}_{2}$peak indicates that either the soccer teams have prioritized other physical qualities for the pre-season or the soccer players possess a $\dot{\text{V}}{\text{O}}_{2}$peak high enough to meet the game’s demands and do not need more aerobic training. A higher MEV could also be due to an increased running capacity and/or an increased running economy, which could be achieved through specific training sessions during the season [19]. However, since the increase in relative $\dot{\text{V}}{\text{O}}_{2}$peak was likely driven more by a decrease in body weight than an increase in aerobic fitness, this underlines that any seasonal change in relative $\dot{\text{V}}{\text{O}}_{2}$peak [20, 21] should be controlled for changes in body weight (Figure 5).

As the present study is based on real-world settings, there are some limitations that are typical of contexts dealing with top-level athletes. For example, the athletes stand quietly on the treadmill for only one minute before commencing the exercise instead of waiting at least 5 minutes as per standard procedures. This was due to the limited time available with the athletes. We could not obtain most of the athletes’ peripheral blood samples at the end of the exercise; therefore, we could not control the lactate concentration at exhaustion. On the other hand, the strength of the present study is that the findings are robust, considering the large dataset with over 700 data points. To ascertain whether the herein-reported level of $\dot{\text{V}}{\text{O}}_{2}$peak would be necessary for reaching the first league, future research should compare the present results with lower leagues.

## CONCLUSIONS

This study contributes to the understanding of the physical and physiological profile of elite soccer players. Our findings from a large data set confirm that $\dot{\text{V}}{\text{O}}_{2}$peak values ~62 ml/kg/min fulfil the demands for $\dot{\text{V}}{\text{O}}_{2}$peak in men’s professional soccer. While playing position did not affect absolute $\dot{\text{V}}{\text{O}}_{2}$peak, the midfielders had the highest relative $\dot{\text{V}}{\text{O}}_{2}$peak and lowest body mass. Similarly, the season phase did not affect absolute $\dot{\text{V}}{\text{O}}_{2}$peak, but the players had the highest relative $\dot{\text{V}}{\text{O}}_{2}$peak and the lowest body mass during the season.

## Perspectives

The reference values provided in the present study represent a standard against which soccer players can compare their own performance and fitness. This will help coaches to set realistic goals and expectations for improvement of their athletes.
